# A chart review of human immunodeficiency virus status in patients admitted with psychosis in Durban, South Africa

**DOI:** 10.4102/sajpsychiatry.v24i0.1129

**Published:** 2018-04-17

**Authors:** Sellwane M. Mere, Saeeda Paruk

**Affiliations:** 1 Department of Psychiatry, University of KwaZulu-Natal, South Africa

## Abstract

**Background:**

Comorbid human immunodeficiency virus (HIV) infection among patients with psychotic disorders is associated with a poorer outcome. Understanding the association of HIV infection with demographic and clinical variables may provide clues to modify risk factors and outcomes.

**Aim:**

To describe and compare the socio-demographic and clinical profile of patients admitted with psychotic disorders with and without HIV infection.

**Method:**

A retrospective chart review of 100 adult patients consecutively admitted with psychosis and HIV infection and compared to 101 patients with psychosis without HIV infection.

**Results:**

HIV-infected patients with psychotic disorders were more likely to be females (74.0%), younger than 50 years (94.0%) and less likely to have secondary education than HIV- negative patients with psychotic disorders (56.0% vs. 72.0%). HIV-infected patients were also less likely to be diagnosed as having schizophrenia (33.0%), had higher rates of medical (73.0%) and psychiatric (21.0%) comorbid disorders and were less likely to report lifetime nicotine and cannabis use (*p* = 0.047 and *p* = 0.011). HIV-negative patients with psychotic disorders were more likely to be readmitted to the unit in the next 12 months (*p* < 0.05). HIV-infected patients with psychotic disorders had increased abnormal haematological results (33.0%).

**Conclusion:**

Patients with psychotic disorders and HIV infection had several negative prognostic factors such as younger age, increased rates of medical and psychiatric comorbidity, abnormal haematological results and longer length of admission periods. This suggests the need to target HIV prevention programmes at young females with mental illness and provide an integrated healthcare service with medical and psychiatric assessment and care for patients with HIV and psychosis.

## Introduction

Human immunodeficiency virus (HIV) infection and acquired immunodeficiency syndrome (AIDS) remain a challenge to public health providers globally and especially in resource-constrained settings like South Africa (SA).^1^ The joint United Nations Programme on HIV/AIDS (UNAIDS) estimated that 2.3 million new infections and 1.6 million AIDS deaths occurred in 2012 and almost 35.3 million adults and children worldwide are living with HIV or AIDS. Sub-Saharan Africa remains the region worst affected by HIV and AIDS.^2^ SA has the highest number of people living with HIV.^2,3^

The association between HIV and psychosis is complex. HIV infection can cause neuropsychiatric complications, including psychosis, and conversely patients with psychosis are more vulnerable to HIV infection.^4^ A survey of risk behaviour for contracting HIV among adult psychiatric patients at Weskoppies Hospital, SA, reported that mentally ill patients are more vulnerable, as they may be victimised and mental illness may impair appreciation of consequences.^5^

HIV sero-positivity in psychiatric patients ranges up to 29.1%.^6^ A study of sero-positivity in patients with first episode psychosis at Town Hill Hospital (KwaZulu-Natal, SA) in 2007 reported a sero-prevalence rate of 23.8%, and subsequently a study on exposure to trauma and the clinical presentation of first episode psychosis found a similar HIV sero-positivity rate of 22.0% among those who had been tested during their admission.^3,7^ A more recent study in recently admitted and long-term psychiatric in-patients at Weskoppies Hospital (Pretoria, SA) reported an HIV prevalence of 11.0%, with women affected more than men.^8^ This is supported by international literature, which suggests a high prevalence of HIV infection among patients with severe mental illness (up to 29.0%) compared to the general population (0.6% – 11.0%).^6,9^

HIV infection is also associated with psychiatric complications as a result of direct viral infection of the central nervous system (CNS) or secondarily because of the complications of being immune-compromised or from the side effects of the antiretroviral medication.^4^ Psychosis may occur in 0.5% – 15.0% of HIV-positive patients.^10^ A team of researchers in Denmark found that HIV-positive people had between a two- and fourfold elevated risk for developing schizophrenia compared to HIV-negative people and they also had a four- to sevenfold elevated risk for developing psychosis.^11^

There are several challenges in diagnosing a primary psychotic disorder in patients presenting with HIV, as the virus may present with delirium, major neurocognitive disorders or encephalitis. Other challenges include the role of opportunistic CNS infections, medication, psychological stress and comorbid substance use in patients with HIV.^1,4,10^ Psychosis in HIV-infected patients may also be part of a major neurocognitive disorder caused by HIV.^10^

HIV infection is associated with greater morbidity and mortality in patients with schizophrenia than in the general population because this comorbidity complicates help seeking, diagnosis and quality of care provided.^1,3,4,12,13^ Therefore, mental health professionals must identify and modify behavioural factors related to HIV risk.^1,4^

There is limited literature on HIV and psychosis specifically, with previous studies mainly describing HIV prevalence rates or clinical profile.^4,14^ A study of the clinical profile of acute psychiatric in-patients with HIV infection in Gauteng reported that patients were predominantly female, in the 20–40 year age group and black, and psychosis was the most common diagnosis.^14^ A Ugandan study by Lundberg and colleagues reported on HIV prevalence among patients with severe mental illness and found that HIV-positive patients with mental illness were more likely to be female and older (40–49 years).^11,15^ Finally, another local study of HIV sero-positivity in patients with first episode psychosis at Town Hill Hospital, KwaZulu-Natal, reported that patients who tested HIV-positive were less educated.^3^ In contrast Gregson and colleagues showed that more educated people were at greater risk of HIV infection in the early stages of the epidemic but tended to adopt less risky behaviours when faced with the facts of HIV transmission.^16^ Therefore, targeted HIV education plays an important role in prevention of HIV transmission and better education should lead to the adoption of safer sexual behaviour in the long term.^16^

Treatment considerations for prescribing antipsychotics in HIV-positive patients include increased susceptibility of side effects to medication and drug-drug interactions.^13^ Research has demonstrated that HIV-infected individuals are more sensitive to the extrapyramidal side effects.^13^ Thus atypical antipsychotics in low doses are supported over the first generation antipsychotic agents such as chlorpromazine, even though atypicals have their own side effects, like metabolic syndrome.^4,13,17,18,19^ First generation antipsychotic agents are commonly used in resource-constrained settings and they have proven to be safe and effective when used in lower doses, but this requires further study.^19^

Because of this bidirectional link between HIV and psychosis, our study aims to evaluate the association of HIV status on psychosis by comparing the patients admitted with psychosis with and without HIV, in terms of their demographic and clinical profile. Better understanding of the role of HIV infection on factors such as length of admission (LOA), 12-month readmission rate, diagnosis and comorbid disorders will assist to provide data on a highly vulnerable clinical population, increase awareness and ultimately improve the care of psychotic patients with HIV infection.

## Aim

The aim of this study was to describe the profile of patients presenting with psychosis and HIV infection and to compare the socio-demographic and clinical profiles of the psychotic patients with HIV to psychotic patients without HIV infection admitted to a psychiatric hospital in Durban, KwaZulu-Natal.

## Methods

### Sample and procedure

A total of 201 (101 HIV-negative with psychosis and 100 HIV-positive with psychosis) people were identified by a retrospective chart review of consecutive adult patients admitted to King Dinuzulu Hospital Complex with psychosis and confirmed HIV status from 01 January 2011 to 31 December 2013.

Patients were defined as HIV-infected if the HIV enzyme-linked immune-sorbent assay (HIV ELISA) test was positive.

Patients presenting with psychotic symptoms meeting criteria for a diagnostic and statistical manual of mental disorders, 4th edition, text revision (DSM-IV-TR) diagnosis of psychotic disorders or affective disorders with psychotic features, requiring antipsychotic medication and admission, were included in the study.

### Procedure

The principal investigator (S.M.M.) collected demographic data (age, gender, religion, ethnicity, language and educational level); HIV status and clinical data including the DSM-IV-TR diagnosis; investigations and treatment details from the patient hospital records. All patients admitted to the unit are fully assessed using a structured assessment format that includes a substance history report from the patient and family if available. This is then further explored and verified by the treating psychiatrists during the course of admission. There was no missing data for substance use.

### Statistical analysis

Data were analysed using SPSS version 21.0 (SPSS Inc., Chicago, IL, USA) package. Associations between variables were assessed using Pearson’s chi-square tests for categorical variables, whilst *t*-tests or Mann–Whitney U tests were used for continuous numerical variables. The one-sample Kolmogorov–Smirnov test for normality was used. Univariate analyses were performed using non-parametric methods for the non-normal distributions of the dependent variables. Fisher’s exact test was used where appropriate. A *p*-value of 0.05 was considered significant.

## Ethical consideration

Confidentiality of records was maintained and there was no direct contact with participants. Ethics approval was obtained from the Biomedical Research Ethics Committee at the University of KwaZulu-Natal, the provincial department of health and the hospital.

## Results

### Demographics characteristics of the sample

The demographic characteristics of all patients with psychosis compared by HIV status are presented in Table 1. The patients admitted with psychosis (*n* = 201) were predominantly black (80.6%), female (60.2%), in the 18–33 year age group (62.7%) and unemployed (70.2%). The patients with psychosis and HIV infection (*n* = 100) were also predominantly black (94%), female (74.0%), in the 18–33 year age group (58.0%), unemployed (66.0%) and less likely to have secondary education (56.0% vs. 72.0%). Patients with psychosis who were not HIV-infected were predominantly male (53.5%).

**TABLE 1:** Socio-demographic characteristics of patients with psychosis.

Characteristics	HIV-negative *n* = 101 *n* (%)	HIV-positive *n* = 100 *n* (%)	Total *n* = 201 *n* (%)	*p*-value (chi-square) significance
**Gender**				
Male	54.0 (53.5)	26.0 (26.0)	80.0 (39.8)	*p* < 0.01 (15.82)
Female	47.0 (46.5)	74.0 (74.0)	121.0 (60.2)	
**Age**				
18–33 years	68.0 (67.0)	58.0 (58.0)	126.0 (62.7)	*p* < 0.01 (10.65)
34–49 years	18.0 (18.0)	36.0 (36.0)	54.0 (26.9)	
50 years and above	15.0 (15.0)	6.0 (6.0)	21.0 (10.4)	
**Marital status**				
Single	77.0 (76.0)	82.0 (82.0)	159.0 (79.0)	*p* > 0.05 (6.06)
Married	15.0 (15.0)	7.0 (7.0)	22.0 (1.0)	
Divorced	4.0 (4.0)	4.0 (4.0)	8.0 (4.0)	
Widowed	3.0 (3.0)	1.0 (1.0)	4.0 (2.0)	
Living with partner	2.0 (2.0)	6.0 (6.0)	8.0 (4.0)	
**Race**				
Black people	68.0 (67.0)	94.0 (94.0)	162 (80.6)	*p* < 0.01 (24.25)
Indian	22.0 (22.0)	3.0 (3.0)	25.0 (12.4)	
Mixed race	3.0 (3.0)	2.0 (2.0)	5.0 (2.5)	
White people	8.0 (8.0)	1.0 (1.0)	9.0 (4.5)	
**Religion**				
Christianity	82.0 (81.0)	98.0 (98)	180.0 (89.5)	*p* < 0.01 (15.29)
Hinduism	12.0 (12.0)	1.0 (1.0)	13.0 (6.5)	
Islam	6.0 (6.0)	1.0 (1.0)	7.0 (3.5)	
None	1.0 (1.0)	0.0 (0.0)	1.0 (0.5)	
**Language**				
Afrikaans	2.0 (2.0)	0.0 (0.0)	2.0 (1.0)	*p* < 0.01 (28.48)
English	32.0 (32.0)	6.0 (6.0)	38.0 (18.9)	
Zulu	64.0 (63.0)	94.0 (94.0)	158 (78.6)	
None listed	3.0 (3.0)	0.0 (0.0)	3.0 (1.5)	
**Social background**				
Living at institution	3.0 (3.0)	1.0 (1.0)	4.0 (2.0)	*p* > 0.05 (6.99)
Rural area	30.0 (30.0)	46.0 (46.0)	76.0 (37.8)	
Urban area	67.0 (66.0)	53.0 (53.0)	120 (59.7)	
Other	1.0 (1.0)	0.0 (0.0)	1.0 (0.5)	
**Employment status**				
Employed	13.0 (13.0)	17.0 (17.0)	30.0 (14.9)	*p* > 0.05 (2.30)
Unemployed	75.0 (74.0)	66.0 (66.0)	141 (70.2)	
Student	3.0 (3.0)	2.0 (2.0)	5.0 (2.5)	
Disability grant	10.0 (10.0)	15.0 (15.0)	25.0 (12.4)	
**Educational level**				
Primary (Grades 1–7)	19.0 (19.0)	32.0 (32.0)	51.0 (25.4)	*p* < 0.05 (5.98)
Secondary (Grades 8–12)	73.0 (72.0)	56.0 (56.0)	129.0 (64.2)	
Tertiary education	9.0 (9.0)	12.0 (12.0)	21.0 (10.4)	

HIV, human immunodeficiency virus.

### Clinical profile of the sample

The association between HIV status in patients with psychosis and clinical variables such as psychiatric diagnosis, medical comorbidity and substance use is presented in Table 2.

**TABLE 2:** Association between human immunodeficiency virus status and clinical variables (*n* = 201).

Characteristics	HIV status		Chi-square (χ^2^)	*p*	OR (95% CI)
	Positive (*n* = 100) Frequency	Negative (*n* = 101) Frequency			
**Psychosis diagnosis**					
Schizophrenia	33.0	79.0	41.64	**0.000**	**0.14 (0.08–0.26)**
Affective psychosis	10.0	14.0	0.71	0.339	0.69 (0.29–1.64)
Substance-induced psychotic disorder	2.0	4.0	0.67	0.41	0.50 (0.09–2.77)
Psychotic disorder caused by HIV	55.0	4.0	63.12	**0.000**	**29.6 (10.12–82.82)**
**Comorbid disorder**					
Medical disorder†	73.0	24.0	48.79	**0.000**	**8.67 (4.59–16.39)**
Psychiatric disorder	21.0	7.0	8.30	**0.004**	**3.57 (1.44–8.83)**
Substance use disorder	15.0	30.0	6.25	**0.012**	**0.42 (0.21–0.84)**
**Lifetime substance use history**					
Nicotine	22.0	34.7	3.96	**0.047**	**0.53 (0.28–0.99)**
Alcohol	30.0	34.7	0.50	0.481	0.81 (0.45–1.46)
Cannabis	23.0	39.6	6.44	**0.011**	**0.46 (0.25–0.84)**
Whoonga‡	2.0	4.0	0.67	0.683	0.50 (0.09–2.77)

Note: Statistical significant *p*-values in bold.

† , Comorbid medical disorders other than HIV infection: 36% (13) had tuberculosis (TB) (8 pulmonary TB, 2 TB meningitis and 1 TB abdomen), 22% (8) epilepsy, 8% (3) diabetes mellitus, 8% (3) asthma, 5.5% (2) hypertension, 5.5% (2) hypothyroidism, 5.5% (2) oral candidiasis, 5.5% (2) cellulitis and 1 anaemia;

‡ , Whoonga is a local drug containing cannabis and low-grade heroine.

HIV, human immunodeficiency virus; OR, odds ratio; CI, confidence interval.

HIV-negative status was significantly associated with a diagnosis of schizophrenia (*p* < 0.001) and HIV-infected patients with a diagnosis of psychotic disorder caused by another medical condition (*p* < 0.001). Patients who were HIV-negative were more likely to have been diagnosed with schizophrenia (79.0) than patients who were HIV-positive (33.0) (odd ratio [OR] = 0.14 [95% confidence interval {CI} = 0.08–0.26]).

HIV status was associated with comorbid medical disorders (*p* < 0.001), psychiatric disorder (*p* < 0.01) and substance use (*p* < 0.05).

Furthermore, patients who were HIV-positive were more likely to have been diagnosed with a psychotic disorder caused by another medical condition (55.0%) than patients who were HIV-negative (4.0%).

### Association between human immunodeficiency virus status and hospitalisation

The relationship between HIV status of patients with psychosis and their length of both admission and readmission are presented in Table 3. HIV-positive patients with psychosis had slightly longer admission periods than those who were HIV-negative, but this finding was not statistically significant (*p* < 0.05). More HIV-negative patients with psychosis were readmitted to a psychiatric institution within a 12-month period (*p* < 0.05).

**TABLE 3:** Human immunodeficiency virus status, length of admission and number of readmissions.

Variable	HIV-positive (*n* = 100)		HIV-negative (*n* = 101)		*t*
	Mean	s.d.	Mean	s.d.	
Length of admission (weeks)	6.61	4.22	6.12	4.24	0.82 (*p* = 0.41)
Number of readmissions	0.24	0.68	0.40	0.75	1.54 (*p* = 0.025)

HIV, human immunodeficiency virus; s.d., standard deviation.

### Association between human immunodeficiency virus and treatment

The relationship between HIV status of patients with psychosis and side effects of antipsychotics are presented in Table 4. Seventy-six HIV-infected individuals were on antiretroviral therapy (ART) during the admission. All the patients received antipsychotics. The most commonly used antipsychotic was risperidone (an atypical antipsychotic) in patients with psychosis and HIV and chlorpromazine (a typical antipsychotic) in HIV-negative patients with psychosis. The most common side effect reported was extrapyramidal side effects by all patients and this was slightly higher in the HIV-negative patients.

**TABLE 4:** Human immunodeficiency virus status and side effects of antipsychotics (*n* = 201).

Documented side effects	HIV status (%)		Chi-square (χ2)/Fisher’s exact test	*p*
	Positive *n* = 100	Negative *n* = 101		
Extrapyramidal	34.0	38.6	0.46	0.496
Metabolic	0.0	0.0	0.0	0.0
Anticholinergic	4.0	7.9	1.38	0.371
Cardiac	3.0	3.0	0.00	0.990
Sedation	1.0	5.0	2.71	0.100
Other	3.0	9.9	3.96	0.082

HIV, human immunodeficiency virus.

### Human immunodeficiency virus status and diagnostic investigation

The relationship between HIV status and the diagnostic investigations are presented in Table 5. HIV infection was significantly associated with increased rates of abnormal full blood count (*p* < 0.001), liver function test (*p* < 0.001), glucose (*p* < 0.01) and thyroid function test (*p* < 0.05) results. Eighty-eight per cent of HIV-infected patients with psychosis had CD4 count results recorded in the psychiatric chart.

**TABLE 5:** Human immunodeficiency virus status and diagnostic investigation (*n* = 201).

Diagnostics investigation	HIV status		Chi-square (χ2)/Fisher’s exact test	*p*
	Positive	Negative		
**FBC**				
Normal	67.0	89.1	14.37	**< 0.001**
Abnormal	33.0	10.9	-	
Not done	0.0	0.0	-	
**U&E**				
Normal	95.0	98.0	1.37	0.243
Abnormal	5.0	2.0	-	
Not done	0.0	0.0	-	
**LFT**				
Normal	71.0	95.0	20.68	**< 0.001**
Abnormal	29.0	5.0	-	
Not done	0.0	0.0	-	
**Glucose**				
Normal	99.0	87.1	14.64	**0.002**
Abnormal	1.0	1.0	-	
Not done	0.0	11.9	-	
**TFT**				
Normal	93.0	99.0	6.25	**0.044**
Abnormal	6.0	0.0	-	
Not done	1.0	1.0	-	
**Syphilis serology**				
Normal	99.0	94.0	3.74	0.154
Abnormal	00.0	01.0	-	
Not done	1.0	5.0	-	

Note: Statistically significant *p*-values in bold.

HIV, human immunodeficiency virus; FBC, full blood count; LFT, liver function test; TFT, thyroid function test; U&E, urea and electrolytes.

## Discussion

The key findings of this study relate to the differences in the socio-demographic and clinical profiles of psychotic patients, with or without HIV infection, requiring admission to a psychiatric institution. HIV-infected patients with psychosis were more likely to be female, less than 50 years old and have less secondary education. Clinically patients with psychotic symptoms and HIV infection were more likely to be diagnosed as having a psychotic disorder caused by another medical condition than schizophrenia. The HIV-infected group were also more likely to have other comorbid medical and psychiatric disorders, abnormalities on haematological tests and receive a second-generation antipsychotic. More HIV-negative patients with psychosis were readmitted within 12 months to a psychiatric hospital. The patients with psychosis and HIV infection, however, had decreased self-reported nicotine and cannabis use and there were no significant differences relating to HIV status and side effects or length of hospital admission.

### Socio-demographic profile

The socio-demographic and clinical profile of patients with psychosis and HIV in this study is consistent with other studies,^1,4,8,11,12,15,20^ which also reported a high prevalence of HIV infection among female, black patients with severe mental illness,^8,11,14,15^ who are under 50 years of age.^11,14^ This finding has important public health implications, suggesting that all patients attending psychiatric services should be educated about and screened for HIV early. The finding in this study that patients with psychosis and HIV were less likely to have secondary education and were mostly unemployed supports the global view that HIV is a disease of poverty, consistent with studies reporting high prevalence in adults with a lower level of education and in countries with lower income.^16^ Some literature does report that development and better education may lead to increased risk behaviour initially and hence more rapid HIV transmission at the beginning of the epidemic.^16^ However, HIV education remains an important tool to fight the HIV epidemic by targeting behaviour modification and safer sexual practices.^16^

### Clinical features

Diagnosis of new onset psychosis in HIV-infected patients requires the exclusion of other probable causes of psychosis, especially when psychiatric symptoms are of acute onset, atypical in nature or of late onset. It is often a diagnostic challenge to determine if psychosis in HIV-infected patients is part of an underlying primary psychotic disorder, as in schizophrenia or schizoaffective disorder, or whether the new onset of symptoms in HIV-infected individuals is related to a psychotic disorder caused by HIV infection or other medical complications related to HIV such as tuberculosis or lymphoma of the brain.^10^ Psychotic symptoms may also be caused by antiretroviral medication.^10^ In this study the finding that HIV-positive patients with psychosis (55.0%) were more likely to have been diagnosed with a psychotic disorder caused by a general medical condition than schizophrenia (33.0%) is supported by a similar trend in the literature. In support of this trend, a study on the clinical profile of HIV-positive patients admitted to a psychiatric unit in Gauteng, SA, found that 23 patients were diagnosed with psychotic disorder caused by general medical condition and only 2 patients were diagnosed with schizophrenia.^14^

The findings of increased medical comorbidity in this study further support that patients with psychosis and HIV are at risk of poorer clinical outcomes because of their increased comorbid disorders like epilepsy, tuberculosis and meningoencephalitis.^14^ This association with medical comorbidity can lead to a high risk of death because of factors like early cardiovascular disease, late diagnosis and inadequate treatment of comorbidities.^4,20^ Therefore, early and comprehensive assessment and investigation in HIV-infected patients with mental illness is critical to improve outcome.^4^

The use of substances is associated with increased risk of contracting HIV, especially in patients with severe mental illness.^4,12,13,17^ Previous research suggests that HIV-positive patients with psychosis are frequently diagnosed with substance use disorders, which complicate management of both psychosis and HIV.^12^ In contrast, in this study, HIV-positive patients with psychosis reported lower lifetime nicotine and cannabis use. This conflicting finding on substance use may be because of gender differences in substance use patterns.^1,4,12,17,20^ In this study the HIV-positive patients were predominantly female and substance misuse in psychotic patients is generally more common in males.^21^ An unusual finding from the study of HIV sero-positivity in patients with first episode psychosis in KwaZulu-Natal was that none of the HIV-positive patients with psychosis reported a history of substance use.^3^ This may suggest that the role of substance use in HIV transmission in the KwaZulu-Natal context needs to be further investigated.

### Haematological results

A review on HIV infection and the liver found that abnormal liver function test results are common in HIV-infected patients.^22^ Elevated liver enzymes may be associated with high viral load, prolonged exposure to antiretroviral treatment (such as nevirapine, efavirenz and abacavir), alcohol abuse and co-infection with viral hepatitis.^22^ Full blood count abnormalities like anaemia, thrombocytopaenia and neutropaenia are also common in HIV-infected patients because of anaemia associated with chronic disease or immune-compromised state.^23^ Therefore routine laboratory screening is essential and is supported by this study’s findings of an increased prevalence of haematological abnormalities.^23^ The clinical context of HIV infection in patients with severe mental illness should alert clinicians to be more vigilant and consider a comprehensive medical screen for these patients.

### Hospitalisation

LOA and readmission has long been a focus of attention, especially with the growing realisation that healthcare costs need to be contained. Studies show that psychosis is one of the predictors of LOA and that patients with psychosis tend to stay longer in hospitals.^24^ A study of mental illness and length of in-patient stay for Medicaid recipients with HIV and/or AIDS revealed that LOA was longer for HIV-infected patients diagnosed with severe mental illness; however, in this study, HIV status did not influence length of stay.^25^ This suggests that in resource-constrained settings other factors such as hospital resources and psychosocial factors need to be investigated in relation to LOA.

In this study, more HIV-negative patients with psychosis were readmitted within the 12-month period, suggesting poor symptom control and poor short-term outcome in these patients. Readmission rates may be low for HIV-positive patients, as patients may have been readmitted to local hospitals and not referred back to a psychiatric institution, especially if medical complications arose.

### Treatment factors

In terms of treatment, the high proportion of patients (76.0%) on ART was encouraging; however, all HIV-infected patients should be on ART.^12^ Twenty-one million people in Africa were eligible for ART in 2013, but only 7.6 million people were receiving ART as of December 2012 and there is a need to urgently address this treatment gap.^2^ A recent study of associations between HIV and schizophrenia and their effect on HIV treatment outcome in Denmark found that HIV-infected patients who were taking ART had lower risk of being diagnosed with psychosis.^20^ Therefore there is a need to increase access to ART by promoting HIV testing and improving patients’ knowledge about their HIV status.^26^

The antipsychotic medication option selected differed based on HIV status in this study and reflects the fact that clinicians elected to use atypical antipsychotics consistent with treatment guidelines in HIV-positive patients. The prescribing guidelines in psychiatry support the use of atypical antipsychotics as first line in HIV-infected patients with psychosis.^27^ The most common side effect experienced or reported was extrapyramidal side effects by all patients; this was slightly higher in the HIV-negative patients, where typical antipsychotics were most commonly used, and is consistent with the literature and treatment guidelines.^28^

### Limitations of the study

There are several limitations in this study. Limitations include the retrospective design of the study; thus, the data are limited by the quality of hospital records available. This study is also limited by the small sample size, heterogeneous sample with affective and non-affective psychosis and the fact that substance use could not be more precisely defined in terms of patterns of use because of limited record keeping. The hospital-based study may have a biased sample with reference to severity of illness; this also limits generalisability to community samples but is reflective of the current clinical profile of patients admitted to a psychiatric institution. The lack of differentiation between patients with first episode and chronic psychotic disorders may also limit results.

This study, however, provides useful insight into the clinical profile of patients with psychosis and HIV infection requiring admission in a resource-challenged setting.

## Conclusion

This study, comparing the HIV statuses of patients with psychosis, suggests that HIV-infected patients with psychosis were more likely to be female and younger. The findings that patients with HIV infection and psychosis have increased prevalence of comorbid medical and psychiatric disorders, haematological abnormalities and readmission are suggestive of poorer outcomes. The finding of decreased substance use among patients with psychosis and HIV in this study is viewed with caution because of the limited sample size and needs to be further explored. The findings suggest the need for a more integrated healthcare service that addresses medical and psychiatric problems in HIV-infected persons with severe mental illness.
